# Association Between Accelerometer-Assessed Physical Activity and Severity of COVID-19 in UK Biobank

**DOI:** 10.1016/j.mayocpiqo.2021.08.011

**Published:** 2021-08-20

**Authors:** Alex V. Rowlands, Paddy C. Dempsey, Clare Gillies, David E. Kloecker, Cameron Razieh, Yogini Chudasama, Nazrul Islam, Francesco Zaccardi, Claire Lawson, Tom Norris, Melanie J. Davies, Kamlesh Khunti, Tom Yates

**Affiliations:** a Diabetes Research Centre, Leicester General Hospital, University of Leicester, Leicester, United Kingdom; b Leicester Real World Evidence Unit, Diabetes Research Centre, Leicester General Hospital, University of Leicester, Leicester, United Kingdom; c National Institute for Health Research (NIHR) Leicester Biomedical Research Centre (BRC), University Hospitals of Leicester NHS Trust and the University of Leicester, Leicester, United Kingdom; d MRC Epidemiology Unit, Institute of Metabolic Science, University of Cambridge, Cambridge, United Kingdom; e Physical Activity and Behavioural Epidemiology Laboratories, Baker Heart and Diabetes Institute, Melbourne, Australia; f St George’s University of London, Tooting, London, United Kingdom; g Nuffield Department of Population Health, University of Oxford, Oxford, United Kingdom; h NIHR Applied Research Collaboration–East Midlands (ARC-EM), Leicester General Hospital, Leicester, United Kingdom

**Keywords:** COVID-19, coronavirus disease 2019, MVPA, moderate to vigorous physical activity, SARS-CoV-2, severe acute respiratory syndrome coronavirus 2

## Abstract

**Objective:**

To quantify the association between accelerometer-assessed physical activity and coronavirus disease 2019 (COVID-19) outcomes.

**Methods:**

Data from 82,253 UK Biobank participants with accelerometer data (measured 2013-2015), complete covariate data, and linked COVID-19 data from March 16, 2020, to March 16, 2021, were included. Two outcomes were investigated: severe COVID-19 (positive test result from in-hospital setting or COVID-19 as primary cause of death) and nonsevere COVID-19 (positive test result from community setting). Logistic regressions were used to assess associations with moderate to vigorous physical activity (MVPA), total activity, and intensity gradient. A higher intensity gradient indicates a higher proportion of vigorous activity.

**Results:**

Average MVPA was 48.1 (32.7) min/d. Physical activity was associated with lower odds of severe COVID-19 (adjusted odds ratio per standard deviation increase: MVPA, 0.75 [95% CI, 0.67 to 0.85]; total, 0.83 [0.74 to 0.92]; intensity, 0.77 [0.70 to 0.86]), with stronger associations in women (MVPA, 0.63 [0.52 to 0.77]; total, 0.76 [0.64 to 0.90]; intensity, 0.63 [0.53 to 0.74]) than in men (MVPA, 0.84 [0.73 to 0.97]; total, 0.88 [0.77 to 1.01]; intensity, 0.88 [0.77 to 1.00]). In contrast, when mutually adjusted, total activity was associated with higher odds of a nonsevere infection (1.10 [1.04 to 1.16]), whereas the intensity gradient was associated with lower odds (0.91 [0.86 to 0.97]).

**Conclusion:**

Odds of severe COVID-19 were approximately 25% lower per standard deviation (∼30 min/d) MVPA. A greater proportion of vigorous activity was associated with lower odds of severe and nonsevere infections. The association between total activity and higher odds of a nonsevere infection may be through greater community engagement and thus more exposure to the virus. Results support calls for public health messaging highlighting the potential of MVPA for reducing the odds of severe COVID-19.

Poor outcomes from coronavirus disease 2019 (COVID-19) are more likely in people who are older,^1^ are more deprived,^2^ have comorbidities,^3^ or are from ethnic minority populations.^4^ As with chronic disease, research suggests that risk factors related to health behaviors, such as obesity^5^ and slow walking pace,^6^ also have a negative impact on COVID-19 outcomes.

Physical activity is a modifiable health behavior that may mitigate the risks of COVID-19.^7^ This could occur through reductions in chronic inflammation^8^^,^^9^ or cardiometabolic risk factors,^10^ which are associated with an increased risk of COVID-19,^11^ or through enhanced immunity.^7^ In the early months of the pandemic (up to July 2020), we reported initial observations from Biobank data,^12^ which was suggestive evidence for lower odds (up to 20%) of severe COVID-19 per 30 minutes of daily moderate to vigorous physical activity (MVPA; *P*=.06). Consistent with this finding, Sallis et al^13^ reported that being physically inactive is the strongest *modifiable* risk factor for severe COVID-19 and stressed the importance of this message for public health. They found that the risk of hospitalization or death with COVID-19 up to October 2020 in a sample of US health plan members was more than twice as high in people who self-reported consistently being inactive in the 2 years before the pandemic compared with those who self-reported consistently meeting the guidelines of 150 minutes of MVPA per week.^13^

Whereas most of the evidence on associations between physical activity and a wide range of health outcomes has similarly been gleaned from self-report methods,^14^ self-report has well-documented limitations, not least of which is the reliance on recall and consequently a focus on purposeful activity.^14^ Accelerometers directly measure movement, reducing measurement error and facilitating a more nuanced consideration of physical activity (eg, the relative importance of the total amount or intensity of physical activity).^15^^,^^16^ UK Biobank is the largest data set with accelerometer-assessed physical activity, having assessed about 100,000 participants.^17^

In this study, we use UK Biobank data from the first and second waves of the COVID-19 pandemic to determine the association between accelerometer-assessed physical activity (measured between 2013 and 2015) and severe and nonsevere COVID-19 outcomes. We hypothesized that physical activity would be associated with reduced odds of severe COVID-19. However, for nonsevere COVID-19, which reflects community transmission, we hypothesized that associations would be attenuated as higher physical activity levels may also reflect greater exposure to the virus.

## Methods

### Study Design

This is a retrospective observational study. Physical activity was assessed between June 2013 and December 2015, 4 to 7 years preceding the COVID-19 pandemic.

### Setting and Cohort

This study uses data from UK Biobank (Application 36371), a prospective cohort of more than 500,000 adults aged 40 to 69 years with baseline assessments conducted between March 2006 and July 2010.^18^ Some participants took part in further touchscreen interviews between 2009 (n=8503) and 2018 (n=15,140). Methods were carried out in accordance with relevant guidelines and regulations, and all participants gave written informed consent before data collection. UK Biobank has full ethical approval from the NHS National Research Ethics Service (16/NW/0274). Data are linked to national severe acute respiratory syndrome coronavirus 2 (SARS-CoV-2) laboratory test data through Public Health England’s Second Generation Surveillance System and include specimen origin (hospital inpatient vs other). COVID-19 testing data are available for England from March 16, 2020; thus, participants from non-English centers and individuals who had died before March 16, 2020, were excluded. Analyses were based on March 16, 2021, refresh data. A flow chart detailing all participant exclusions is provided in Supplemental Figure 1 (available online at http://mcpiqojournal.org).

### COVID-19 Outcomes

Two outcomes were reported: severe infection with SARS-CoV-2 and nonsevere infection with SARS-CoV-2. A positive test result for SARS-CoV-2 with hospitalization or death related to the disease (ie, any death with an *International Statistical Classification of Diseases, Tenth Revision* code of U07.1 or U07.2 as the primary cause of death on the death certificate) was considered evidence of a severe infection.^19^ A positive test result for SARS-CoV-2 from a community setting without a hospital diagnosis or death was considered evidence of probable mild disease or a nonsevere infection. Classifying a positive test result for SARS-CoV-2 in those admitted to the hospital as a marker of disease severity within UK Biobank is in line with guidance for this data set.^19^ However, actual disease severity cannot be confirmed from the linkage data available. Therefore, our reference to the composite of a test result for SARS-CoV-2 in those admitted to the hospital or death from COVID-19 as indicating “severe” disease is for descriptive purposes only.

### Physical Activity

A subsample of approximately 100,000 adults were asked to wear the Axivity AX3 wrist-worn accelerometer 24 hours a day for 7 days between June 2013 and December 2015.^17^ For each participant, we extracted the accelerometer data (5-second epoch time series) from UK Biobank^17^ and converted it to R-format for processing and analysis with GGIR (version 1.11-0; http://cran.r-project.org).^20^ Participants were excluded if they failed calibration, they had fewer than 3 days of valid wear (defined as >16 h/d), or wear data were not present for each 15-minute period of the 24-hour cycle.^12^^,^^15^^,^^21^ Accelerometer outcomes, selected to describe total physical activity and its intensity, were as follows:

• average acceleration during the 24-hour day (proxy for total physical activity, m*g*);
• intensity gradient during 24 hours (intensity distribution of activity during the day; higher values indicate that a greater proportion of total activity is spent at high intensity)^21^; and
• time spent in 1-minute bouts of MVPA (acceleration cut point 100 m*g*^22^).

### Statistical Analyses

Logistic regression was used to analyze associations of physical activity with the COVID-19 outcomes:

Model 1: severe COVID-19 infection (N=434) with no test or negative test result (N=79,856) as comparator
Model 2: severe COVID-19 infection (N=434) with nonsevere infection (N=1963) as comparator
Model 3: nonsevere COVID-19 infection (N=1963) with no test or negative test result (N=79,856) as comparator

Models 1 and 3 (no test or negative test result as comparator) can be interpreted as the increased or decreased odds of being admitted to the hospital or dying of COVID-19 (severe disease; model 1) or of having a positive test result in the community (nonsevere disease; model 3) during the linkage period within UK Biobank. Model 2 can be interpreted as the risk of any positive test result being from a setting (hospital) or outcome (death) that indicates severe disease during the linkage period within UK Biobank. Assessing the odds of infection relative to the cohort (models 1 and 3) is commonly reported within COVID-19 risk factor research and enables comparison to the literature in terms of how the risk factors assessed compare with other commonly reported risk factors (eg, obesity^5^).

Three physical activity exposures were considered: total physical activity, intensity gradient, and MVPA. A mutually adjusted model was also run for total physical activity and the intensity gradient to test whether associations were independent of the alternative activity metric consistent with previous research assessing the relative contributions of total activity and intensity of activity for health.^15^^,^^23^ The variables were standardized before entry into the models and the odds ratios per cohort standard deviation reported for ease of comparison across exposures.^12^ Covariates were selected on the basis of current clinical knowledge and included age (at censoring), follow-up time (difference between age at accelerometer measure and age at censoring), season of accelerometer wear, sex, ethnicity (White, South Asian, Black and African or Caribbean), Townsend score (area-level measure of deprivation), employment status, and cardiovascular disease or cancer diagnosis before accelerometer baseline (self-reported history of heart attack, angina, stroke, or cancer variables or hospital episode with *International Statistical Classification of Diseases, Tenth Revision* code I20-25, I60-69, or C00-99). Additional health-related covariates potentially on the causal pathway from physical activity to COVID-19 risk were included in sensitivity analyses. These were body mass index, blood pressure or cholesterol medication, and prescribed insulin medication. Covariates from the assessment closest to the accelerometer time point were used.

Analyses were reported for the full population and stratified by sex. Effect modification by sex was tested using an interaction term (sex∗physical activity) in the model.

Statistical significance was set at a *P* value of less than .05; results are reported with a 95% CI. Interactions were considered significant at a *P* value of less than .1. All analyses were performed in Stata version 16.1 (StataCorp LLC).

### Sensitivity Analyses

All models were further adjusted for covariates potentially on the causal pathway from physical activity to COVID-19 risk.

As testing in the United Kingdom has not been universal, particularly in the first wave of the pandemic, there is a risk of selection bias in models 1 and 3, where those with COVID-19 were compared with those with a negative test result or no test. Participants with COVID-19 may not have been tested and thus wrongly allocated to the comparator group. To address this, we carried out sensitivity analyses for models 1 and 3, restricting the comparator group to those who tested negative for COVID-19.

## Results

Data from 82,253 UK Biobank participants with accelerometer data (2013-2015) and complete covariate and linked COVID-19 data to March 16, 2021, were included. Of these participants, 12,713 (15.5%) had been tested for COVID-19, 2388 (2.9%) had a positive test result, 1963 (2.4%) were classified as having a nonsevere infection, and 425 (0.5%) were classified as having a severe infection; another 9 (0.01%) were classified as severe without a positive test result. Participants accumulated a mean of 48.1 (SD 32.7) minutes of MVPA per day. Participants’ characteristics are reported in the Table.

**Table:** Participants’ Characteristics by COVID-19 Status^a^^,^^b^

Characteristic	No COVID-19			Severe COVID-19			Nonsevere COVID-19		
	All	Men	Women	All	Men	Women	All	Men	Women
No. [%]	79,856 [100]	34,614 [43.4]	45,242 [56.7]	434 [100]	250 [57.6]	184 [42.4]	1963 [100]	812 [41.4]	1151 [58.6]
Age at census (y)	68.0 (7.77)	68.7 (7.83)	67.6 (7.70)	68.5 (8.73)	69.5 (8.85)	67.2 (8.4)	63.5 (7.72)	64.2 (8.18)	63.0 (7.35)
Follow-up time (y)	5.6 (0.70)	5.6 (0.70)	5.6 (0.70)	5.5 (0.76)	5.5 (0.80)	5.6 (0.69)	5.6 (0.68)	5.6 (0.68)	5.6 (0.68)
Body mass index (kg/m^2^)	26.6 (4.54)	27.2 (4.03)	26.2 (4.85)	28.7 (5.07)	28.7 (4.40)	28.6 (6.01)	27.1 (4.66)	27.7 (4.12)	26.6 (4.95)
Townsend score^c^	−1.7 (2.79)	−1.8 (2.79)	−1.7 (2.79)	−1.4 (3.00)	−1.1 (3.14)	−1.7 (2.79)	−1.3 (2.94)	−1.4 (2.85)	−1.2 (2.99)
Ethnicity									
White	78,470 [98.3]	33,969 [98.1]	44,501 [98.4]	416 [95.9]	239 [95.6]	177 [96.2]	1901 [96.8]	788 [97.0]	1113 [96.7]
South Asian	657 [0.8]	363 [1.1]	294 [0.7]	8 [1.8]	6 [2.4]	2 [1.1]	30 [1.5]	16 [2.0]	14 [1.2]
Black	729 [0.9]	282 [0.8]	447 [1.0]	10 [2.3]	5 [2.0]	5 [2.7]	32 [1.6]	8 [1.0]	24 [2.1]
In paid employment or self-employed	43,206 [54.1]	19,025 [55.0]	24,181 [53.5]	234 [53.9]	123 [49.2]	111 [60.3]	1422 [72.4]	592 [72.9]	830 [72.1]
Prevalent CVD or cancer at baseline^d^	14,105 [17.7]	6806 [19.7]	7299 [16.1]	110 [25.4]	74 [29.6]	36 [19.6]	290 [14.8]	139 [17.1]	151 [13.1]
Blood pressure or cholesterol medication	20,978 [26.3]	11,714 [33.9]	9264 [20.5]	181 [41.7]	124 [49.6]	57 [31.0]	386 [19.7]	215 [26.5]	171 [14.9]
Insulin medication	3154 [4.0]	1949 [5.6]	1205 [2.7]	43 [9.9]	31 [12.4]	12 [6.5]	79 [4.0]	40 [4.9]	39 [3.4]
Physical activity									
Total physical activity (m*g*)	28.3 (8.33)	27.8 (8.66)	28.7 (8.04)	26.5 (8.86)	26.2 (9.27)	27.0 (8.29)	29.9 (8.51)	29.6 (9.09)	30.1 (8.08)
Intensity gradient	−2.55 (0.193)	−2.51 (0.201)	−2.57 (0.182)	−2.59 (0.213)	−2.54 (0.209)	−2.64 (0.206)	−2.52 (0.194)	−2.48 (0.207)	−2.55 (0.179)
MVPA (minutes)	48.1 (32.74)	48.9 (32.85)	47.6 (32.65)	40.0 (33.61)	42.3 (35.46)	37.0 (30.75)	51.7 (34.53)	52.7 (34.96)	51.0 (34.23)

a CVD, cardiovascular disease; MVPA, moderate to vigorous physical activity.

b Values are reported are mean (standard deviation) or number [percentage].

c Composite measure of deprivation based on unemployment, non–car ownership, non–home ownership, and household overcrowding; negative values represent less deprivation.

d Self-reported history of heart attack, angina, stroke, or cancer variables or hospital episode with *International Statistical Classification of Diseases, Tenth Revision* code I20-25, I60-69, or C00-99.

### Severe COVID-19 Relative to No COVID-19 (Model 1) and Relative to Nonsevere COVID-19 (Model 2)

**Figure 1:**
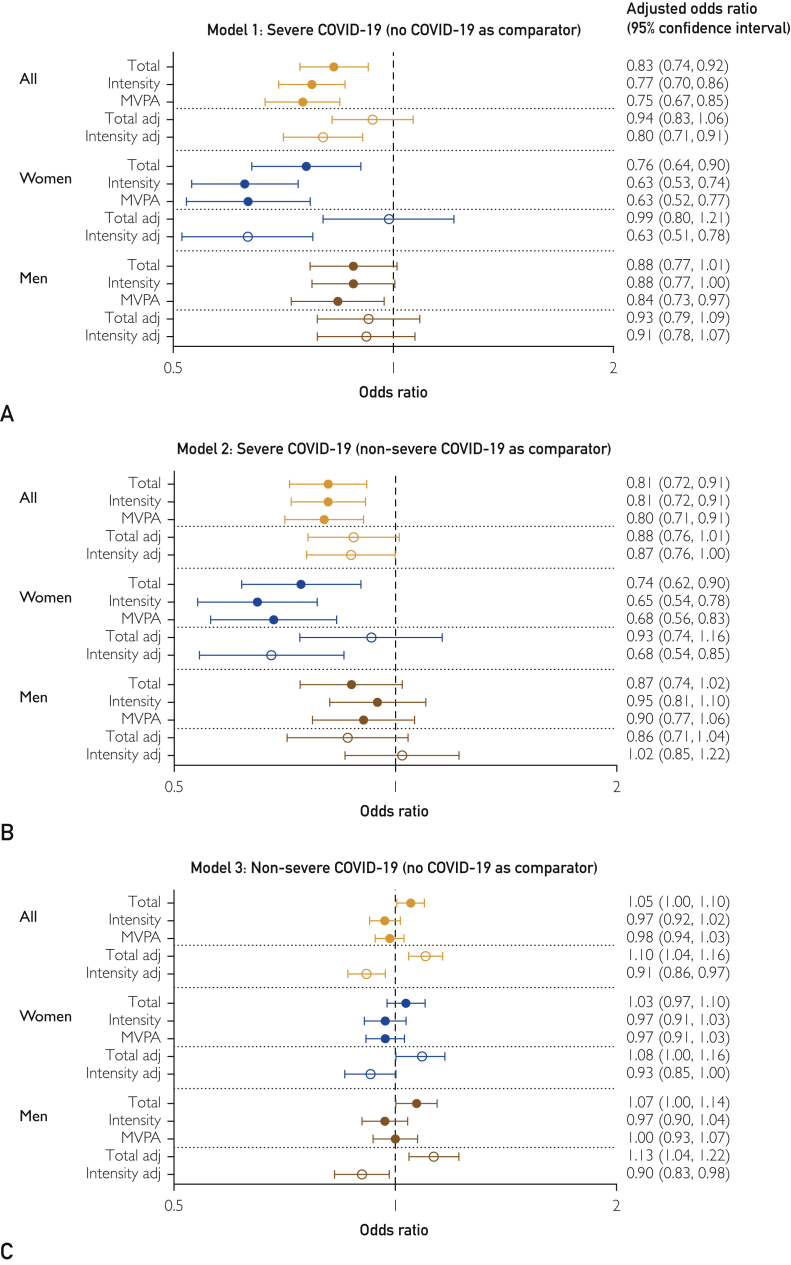
Association of total physical activity, intensity gradient, and moderate to vigorous physical activity (MVPA). A, Model 1, severe COVID-19 (no COVID-19 as comparator). B, Model 2, severe COVID-19 (nonsevere COVID-19 as comparator). C, Model 3, nonsevere COVID-19 (no COVID-19 as comparator). Odds ratios are expressed per standard deviation of each variable. Where *adj* follows the variable name, it indicates the 2 variables were mutually adjusted.

Total physical activity (adjusted odds ratio per standard deviation increase: 0.83 [95% CI, 0.74 to 0.92]), intensity gradient (0.77 [0.70 to 0.86]), and time in MVPA (0.75 [0.67 to 0.85]) were associated with lower odds of a severe infection relative to the cohort (Figure 1A). When mutually adjusted, the intensity gradient remained significant (0.80 [0.71 to 0.91]), but the association with total physical activity was attenuated.

Interactions between sex and physical activity were significant for the intensity gradient (*P*=.03) and MVPA (*P*=.10). Associations were stronger in women for all exposures (total physical activity, 0.76 [0.64 to 0.90]; intensity gradient, 0.63 [0.53 to 0.74]; MVPA, 0.63 [0.52 to 0.77]) than in men (total, 0.88 [0.77 to 1.01]; intensity gradient, 0.88 [0.77 to 1.00]; MVPA, 0.84 [0.73 to 0.97]; Figure 1A).

Results for a severe infection relative to those with a nonsevere infection (model 2) were consistent with those for model 1 (Figure 1B).

### Nonsevere COVID-19 Relative to No COVID-19 (Model 3)

There was an association between total physical activity and higher odds of a nonsevere infection (1.05 [1.00 to 1.10]; *P*=.03; Figure 1C). When mutually adjusted, the intensity gradient was associated with lower odds of a nonsevere infection (0.91 [0.86 to 0.97]), whereas total physical activity was associated with higher odds (1.10 [1.04 to 1.16]). No associations with MVPA were evident. The pattern of results was consistent for men and women.

**Figure 2:**
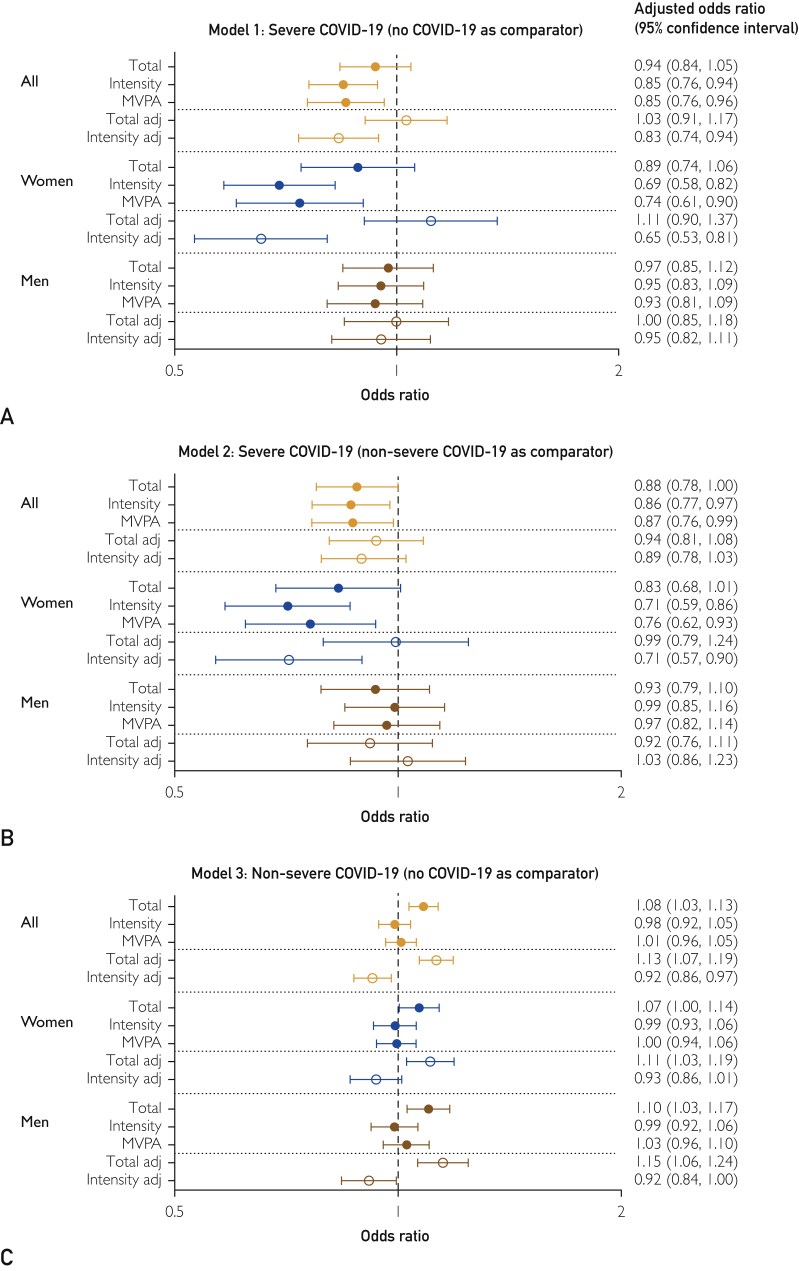
Sensitivity analysis with models further adjusted for health-related covariates potentially on the causal pathway. Association of total physical activity, intensity gradient, and moderate to vigorous physical activity (MVPA). A, Model 1, severe COVID-19 (no COVID-19 as comparator). B, Model 2, severe COVID-19 (nonsevere COVID-19 as comparator). C, Model 3, nonsevere COVID-19 (no COVID-19 as comparator). Odds ratios are expressed per standard deviation of each variable. Where *adj* follows the variable name, it indicates the 2 variables were mutually adjusted.

Sensitivity analyses with further adjustment for health-related covariates potentially on the causal pathway from physical activity to COVID-19 risk (Figure 2) slightly attenuated the effect estimates for models 1 and 2 but were broadly consistent with the main analyses. Models 1 and 3, with the comparator group restricted to people who tested negative for COVID-19 (Supplemental Figure 2, available online at http://mcpiqojournal.org; Supplemental Figure 3, further adjusted for covariates potentially on the causal pathway, available online at http://mcpiqojournal.org), and results of unadjusted models (Supplemental Figure 4, available online at http://mcpiqojournal.org) were broadly consistent with results of the main analyses. Adjusted odds ratios for the risk factors included in the analyses are shown in Supplemental Figures 5 and 6 (available online at http://mcpiqojournal.org).

## Discussion

### Main Findings

Higher physical activity was associated with reduced odds of severe COVID-19; intensity of physical activity was the driving factor, with 20% to 25% lower odds per 30 minutes of daily MVPA (eg, walking). Associations were stronger in women, with 32% to 37% lower odds per 30 minutes of daily MVPA relative to 10% to 16% lower odds in men. Total physical activity appeared to increase the odds of nonsevere COVID-19. As the incidence of nonsevere infections reflects community transmission, this finding probably reflects greater exposure to the virus. In contrast, when adjusted for total activity, a greater proportion of high-intensity activity was associated with 7% to 10% lower odds of infection.

Increased odds of severe COVID-19 with lower total activity and MVPA is consistent with the recent findings from self-reported physical activity in the United States^13^ and from UK Biobank early in the pandemic.^12^ It is not clear why associations with severe COVID-19 tended to be weaker in men than in women for metrics reflecting the intensity of physical activity. Men are known to be at higher risk of severe COVID-19 than women, and although most studies included sex in their analyses as a potential confounder, relatively few studies have reported whether associations with risk factors differ by sex (ie, whether sex is an effect modifier). However, Gao et al^24^ recently reported no difference in associations between body mass index and COVID-19 severity by sex. Conversely, higher odds of severe COVID-19 have been reported for women who work shifts outside of health care (2.77 [2.14 to 3.59]) than for men who work shifts outside of health care (1.59 [1.23 to 2.05]).^25^

### Total Amount and Intensity of Activity

The availability of accelerometer-assessed physical activity enabled us to explore whether the total amount of physical activity and the intensity of that activity were associated with COVID-19 outcomes independent of each other. Independent associations for the intensity gradient for the whole cohort and women for severe COVID-19, alongside reduced odds for MVPA, were observed. This suggests that the proportion of activity taken at a moderate to vigorous intensity is key (eg, walking and brisk walking), consistent with self-report of meeting physical activity guidelines^13^ or having a brisk walking pace.^6^ As time spent in MVPA is associated with cardiorespiratory fitness, this is consistent with evidence that cardiorespiratory fitness^26^ is associated with reduced risk of hospitalization due to COVID-19.^27^^,^^28^

Low levels of physical activity contribute to chronic disease^10^ and chronic inflammation,^9^ which could be a factor in the observed association with severe COVID-19.^11^ Given that COVID-19 is an acute inflammatory disease, inactivity may also exacerbate existing chronic inflammation and, alongside other risk factors (eg, genetic predisposition, psychological factors), be associated with a “cytokine storm” contributing to this increased risk of severe COVID-19.^9^

For nonsevere infections, when mutually adjusted, the intensity gradient was again independently associated with reduced odds of infection, but total physical activity was associated with elevated odds. MVPA, which combines moderate- and vigorous-intensity activity, was not associated with reduced odds. This suggests that intensities greater than moderate (eg, vigorous activity such as brisk walking and running) may be optimal to reduce the odds of a nonsevere infection. It is possible that the observed association between total physical activity and increased odds of a nonsevere infection is due to a bias in the people who were more likely to be tested for COVID-19.^29^ However, results were consistent in restricting the comparator group to people who tested negative for COVID-19. Accumulating evidence indicates that occupation type is associated with COVID-19 outcomes, with elevated risk in essential workers.^30^ Notably, approximately three-quarters of the people with nonsevere COVID-19 were employed, relative to approximately half of the cohort. It is possible that high levels of total physical activity accumulated at relatively low intensities reflect working in a job that requires high levels of routine movement/walking and more community engagement and thus increased exposure to the virus. Whereas we controlled for employment status and deprivation, it was not possible to determine the level of exposure to infection. Conversely, vigorous-intensity activity may reflect exercise or training-type activity that strengthens immunity.^7^

### Strengths and Limitations

The main strength of this study is the availability of accelerometer-assessed physical activity, facilitating precise characterization of the physical activity profile of participants in a large population with linked COVID-19 data. In addition, UK Biobank is an extensively phenotyped population, differentiating it from many other data sets currently being analyzed to better understand COVID-19. However, there are also some limitations. First and foremost, the characteristics of participants were measured—and accelerometer data collected—some years before the pandemic. However, data from a longitudinal study of older adults in England^31^ found steady physical activity levels over time, albeit with a slight decline in vigorous activity. Second, the definition of severe COVID-19 was a positive test result from a hospital inpatient. Whereas this is consistent with the definition proposed by the researchers who developed the linkage method^19^ and with previous research using the UK Biobank data set to explore risk factors for COVID-19,^2^^,^^5^^,^^6^^,^^12^^,^^30^ actual disease severity cannot be confirmed from the linkage data available. Testing in the UK has not been universal, particularly in the first wave of the pandemic, making the analyses vulnerable to bias.^29^ Furthermore, this is an observational study; thus, we cannot exclude the risk of residual confounders due to unmeasured confounders or measurement error. Finally, UK Biobank participants are not representative of the wider population; however, participants may not need to be representative in estimating relative risk factor associations.^32^ Taking these limitations into consideration, our results point to the potential importance of physical activity as predictive of later risk of severe and nonsevere COVID-19 infection. Notably, however, the results are consistent with proposed mechanisms and the reduced risk of severe COVID-19 in participants who self-reported consistently meeting physical activity guidelines in the 2 years before the pandemic.^13^

## Conclusion

Physical activity appeared to be associated with lower odds of severe COVID-19, with stronger associations for intensity of movement in women than in men. Odds of severe COVID-19 were lower by 37% in women and 16% in men per 30 minutes of daily MVPA. Higher total physical activity appeared to increase the odds of nonsevere infection, but a greater proportion of high-intensity activity was associated with 8% to 10% lower odds. Nonsevere infections reflect community transmission; thus, the greater odds associated with higher total physical activity levels, accumulated at lower intensity, are likely to reflect greater exposure to the virus (eg, through occupational activity).

Results from this study are consistent with self-reported data and provide further evidence for the role of physical activity in reducing the odds of a severe infection and the potential for vigorous activity to play a role in reducing the odds of nonsevere infection, possibly through more robust immunity. This is important as a reduction in nonsevere infections has potential for reducing community transmission. This study provides further support to calls for public health messaging to highlight the potential of physical activity, particularly of moderate to vigorous intensity, in reducing the risk of severe COVID-19.
