# Supplementary figures and images for: Association Between Accelerometer-Assessed Physical Activity and Severity of COVID-19 in UK Biobank
Source: Mayo Clin Proc Innov Qual Outcomes. 2021 Aug 20;5(6):997–1007. doi: 10.1016/j.mayocpiqo.2021.08.011 (PMC8376658; doi:10.1016/j.mayocpiqo.2021.08.011)

**Figure S1:** Flow chart of participants included in the study

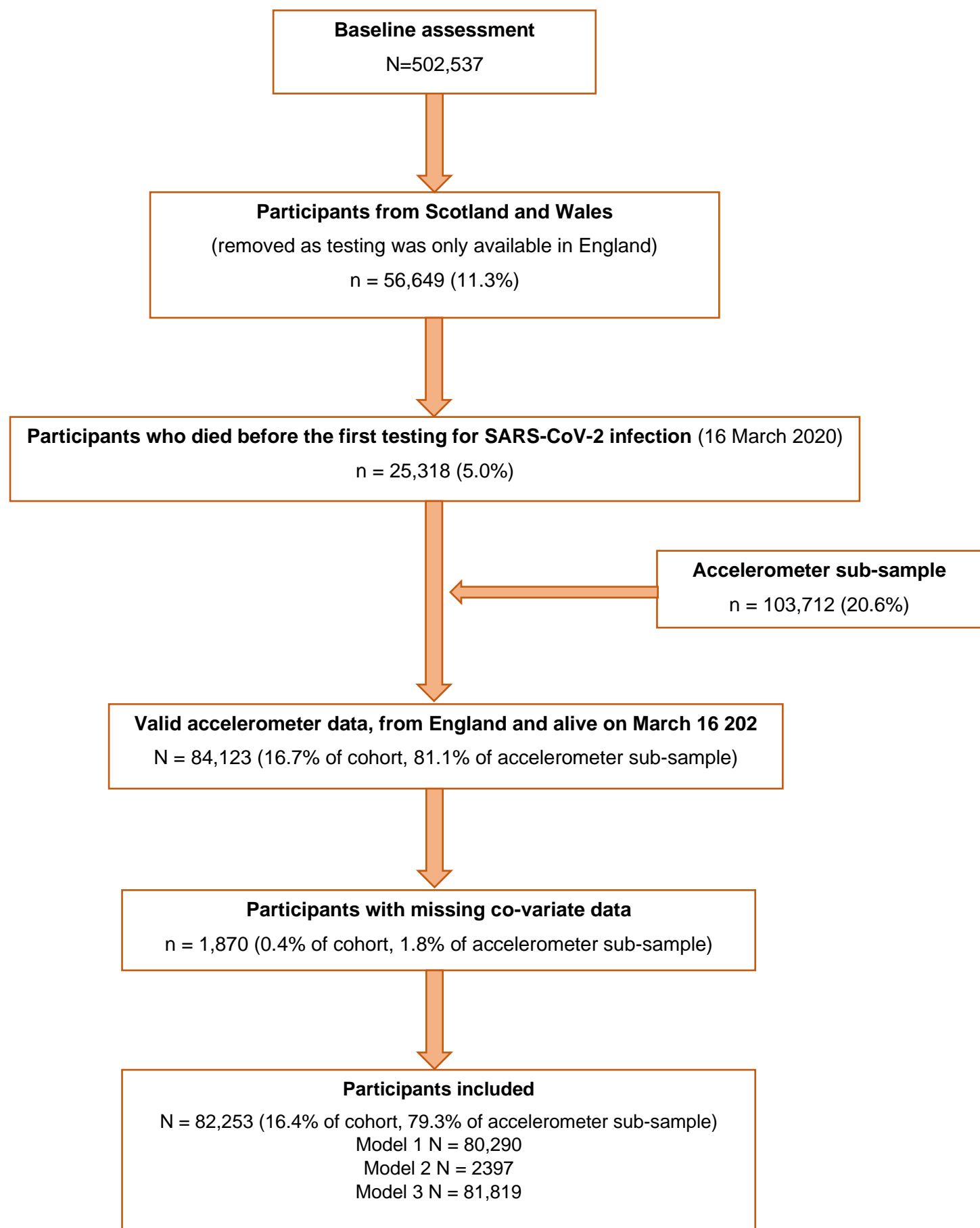

Supplement: Figure S1 [file mmc1.pdf]
