# Supplementary material for: Association Between Accelerometer-Assessed Physical Activity and Severity of COVID-19 in UK Biobank
Source: Mayo Clin Proc Innov Qual Outcomes. 2021 Aug 20;5(6):997–1007. doi: 10.1016/j.mayocpiqo.2021.08.011 (PMC8376658; doi:10.1016/j.mayocpiqo.2021.08.011)
Supplement: Figure S2 [file mmc2.pdf]

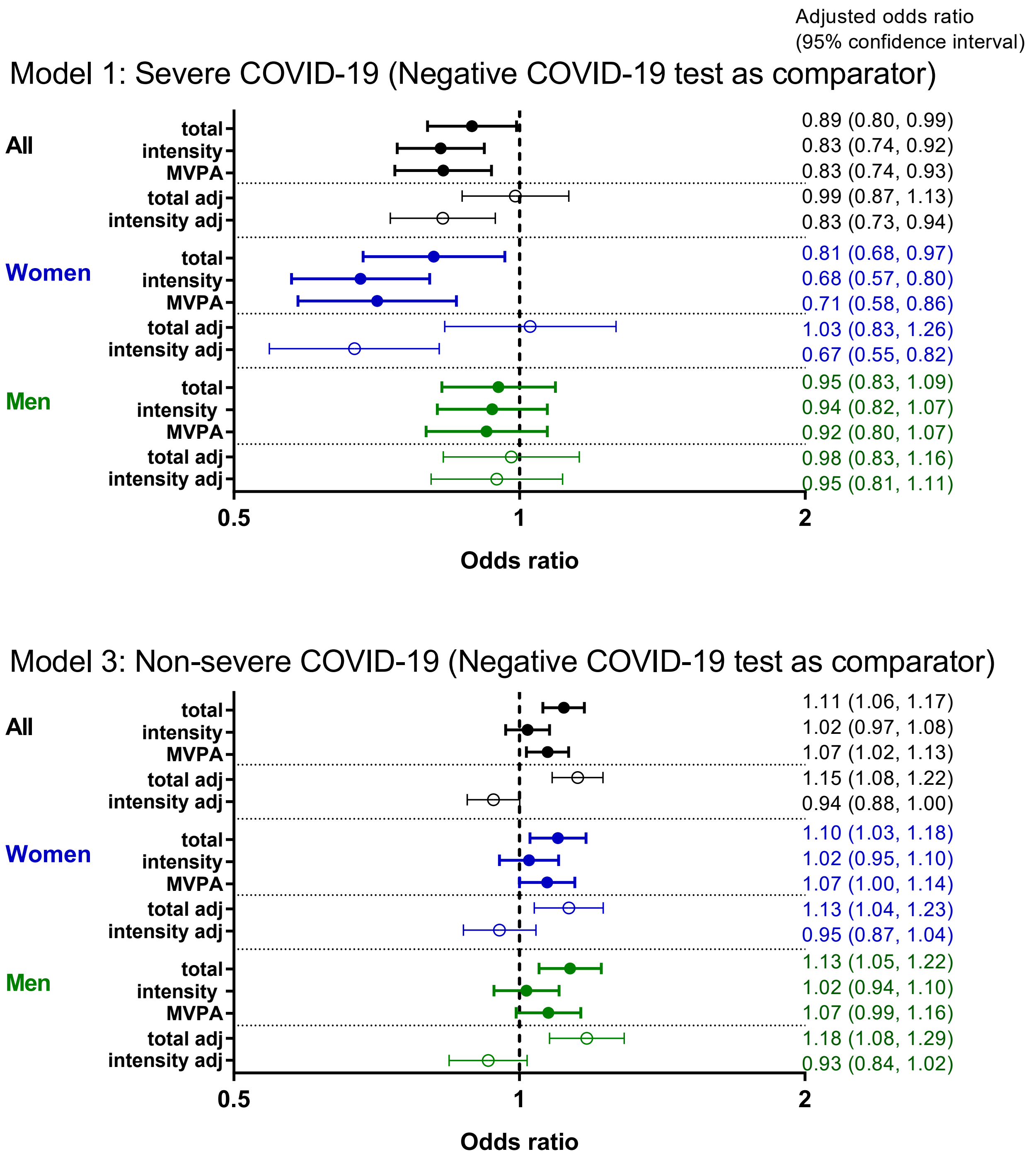

Figure S2. Sensitivity analyses of Models 1 and 3 with negative COVID-19 test as comparator: Association of total physical activity, the intensity gradient, and MVPA with severe and non-severe COVID-19 (negative test as comparator). Odds ratios expressed per standard deviation of each variable. MVPA: moderate-to-vigorous physical activity Where 'adj' follows the variable name, it indicates the two variables were mutually adjusted.
