# Supplementary material for: Association Between Accelerometer-Assessed Physical Activity and Severity of COVID-19 in UK Biobank
Source: Mayo Clin Proc Innov Qual Outcomes. 2021 Aug 20;5(6):997–1007. doi: 10.1016/j.mayocpiqo.2021.08.011 (PMC8376658; doi:10.1016/j.mayocpiqo.2021.08.011)
Supplement: Figure S4 [file mmc4.pdf]

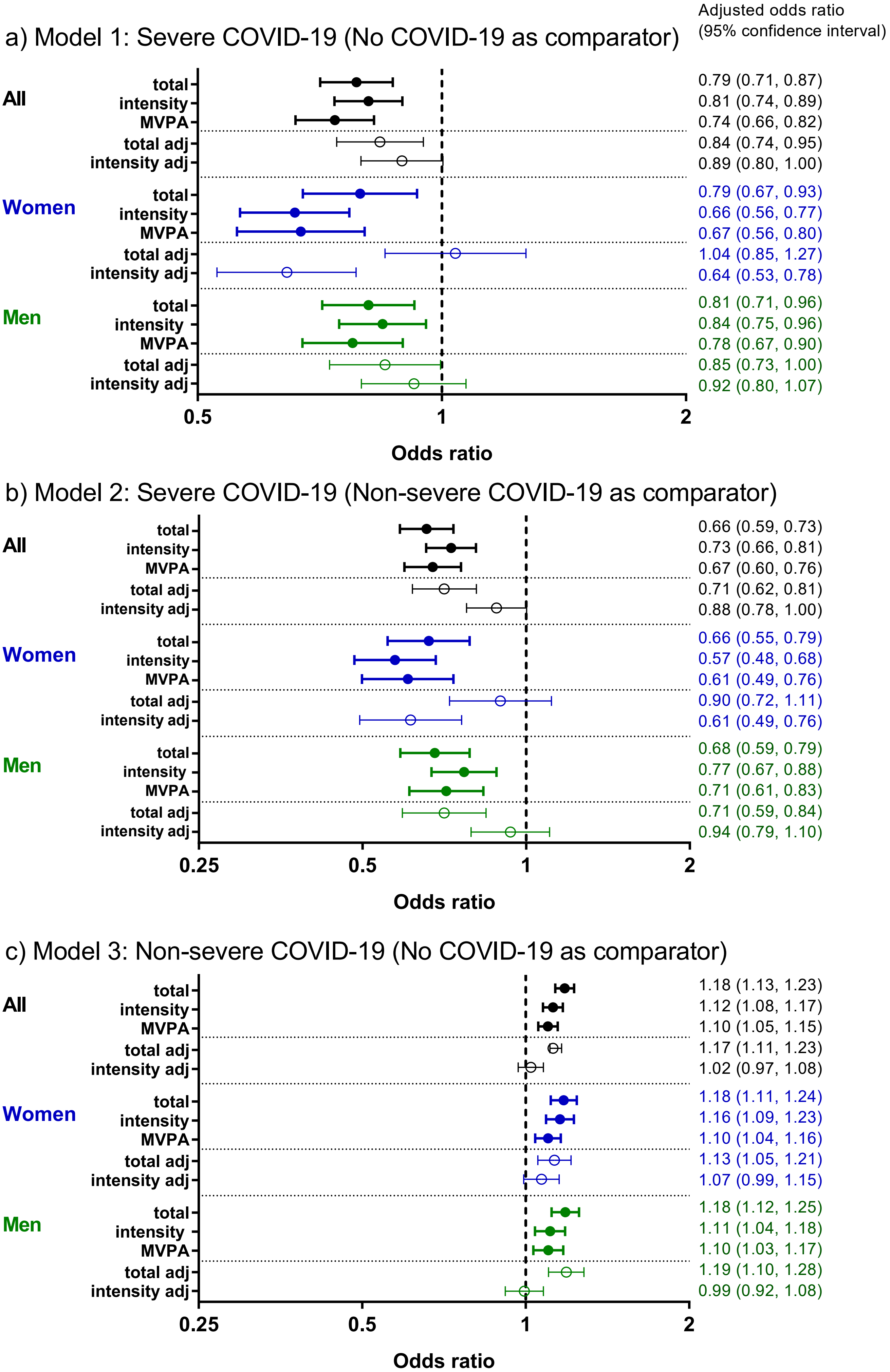

Figure S4. Unadjusted models. Association of total physical activity, the intensity gradient, and MVPA: a) Model 1. Severe COVID-19 (No COVID-19 as comparator); b) Model 2. Severe COVID-19 (Non-severe COVID-19 as comparator); c) Model 3. Non-severe COVID-19 (No COVID-19 as comparator). Odds ratios expressed per standard deviation of each variable. MVPA: moderate-to-vigorous physical activity Where 'adj' follows the variable name, it indicates the two variables were mutually adjusted.
