# Supplementary material for: Association Between Accelerometer-Assessed Physical Activity and Severity of COVID-19 in UK Biobank
Source: Mayo Clin Proc Innov Qual Outcomes. 2021 Aug 20;5(6):997–1007. doi: 10.1016/j.mayocpiqo.2021.08.011 (PMC8376658; doi:10.1016/j.mayocpiqo.2021.08.011)
Supplement: Figure S5 [file mmc5.pdf]

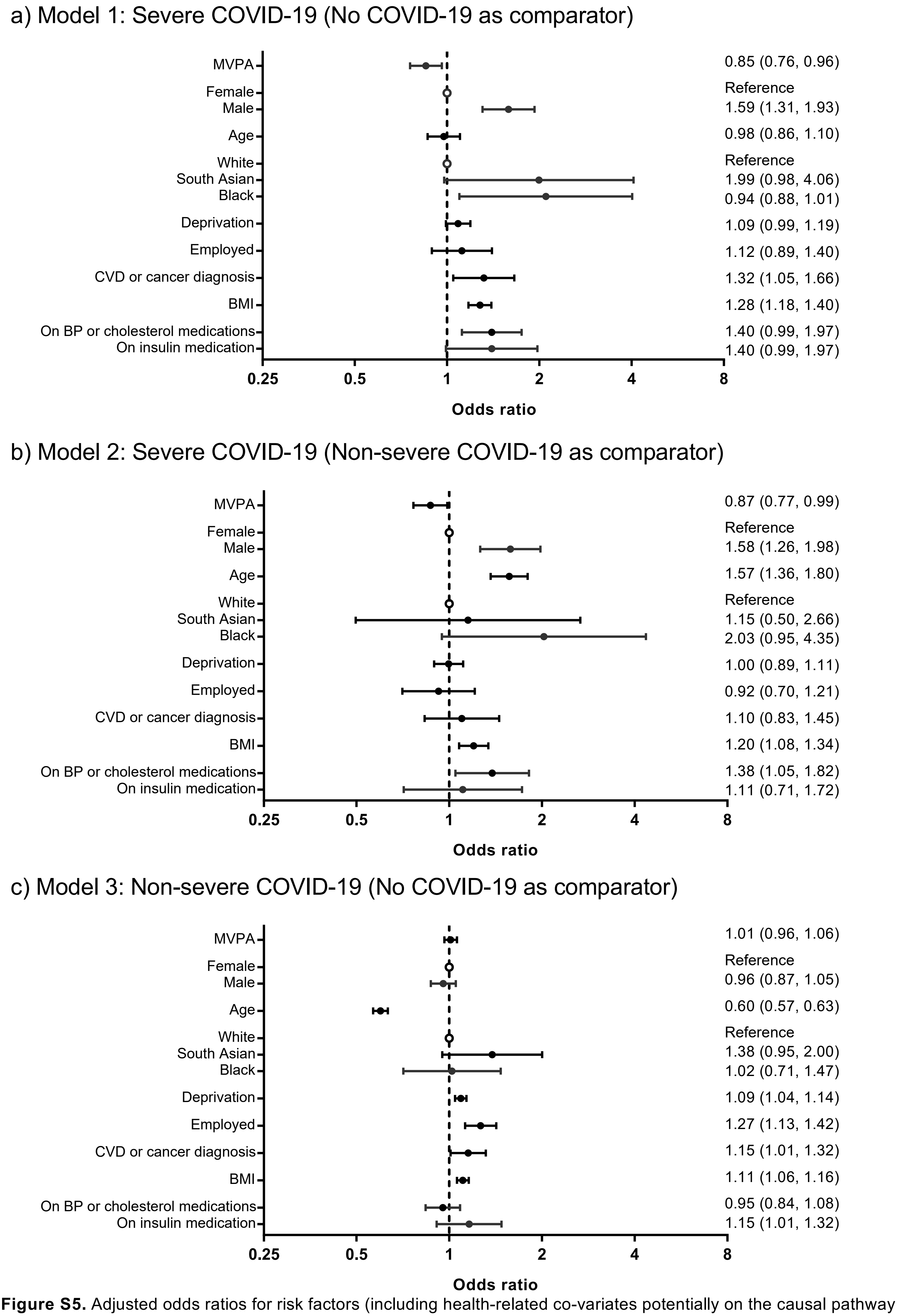

Figure S5. Adjusted odds ratios for risk factors (including health-related co-variates potentially on the causal pathway from physical activity to COVID-19 risk) entered into logistic regression analyses for Models 1, 2 and 3 with MVPA as the exposure. Continuous variables (MVPA, age, BMI and deprivation) are standardized, thus odds ratios expressed per standard deviation. The adjusted odds ratios for co-variates were similar across all physical activity outcomes. Open circles = reference category.
