# Supplementary material for: Association Between Accelerometer-Assessed Physical Activity and Severity of COVID-19 in UK Biobank
Source: Mayo Clin Proc Innov Qual Outcomes. 2021 Aug 20;5(6):997–1007. doi: 10.1016/j.mayocpiqo.2021.08.011 (PMC8376658; doi:10.1016/j.mayocpiqo.2021.08.011)
Supplement: Figure S6 [file mmc6.pdf]

Model 1: Severe COVID-19 (Negative COVID-19 test as comparator)

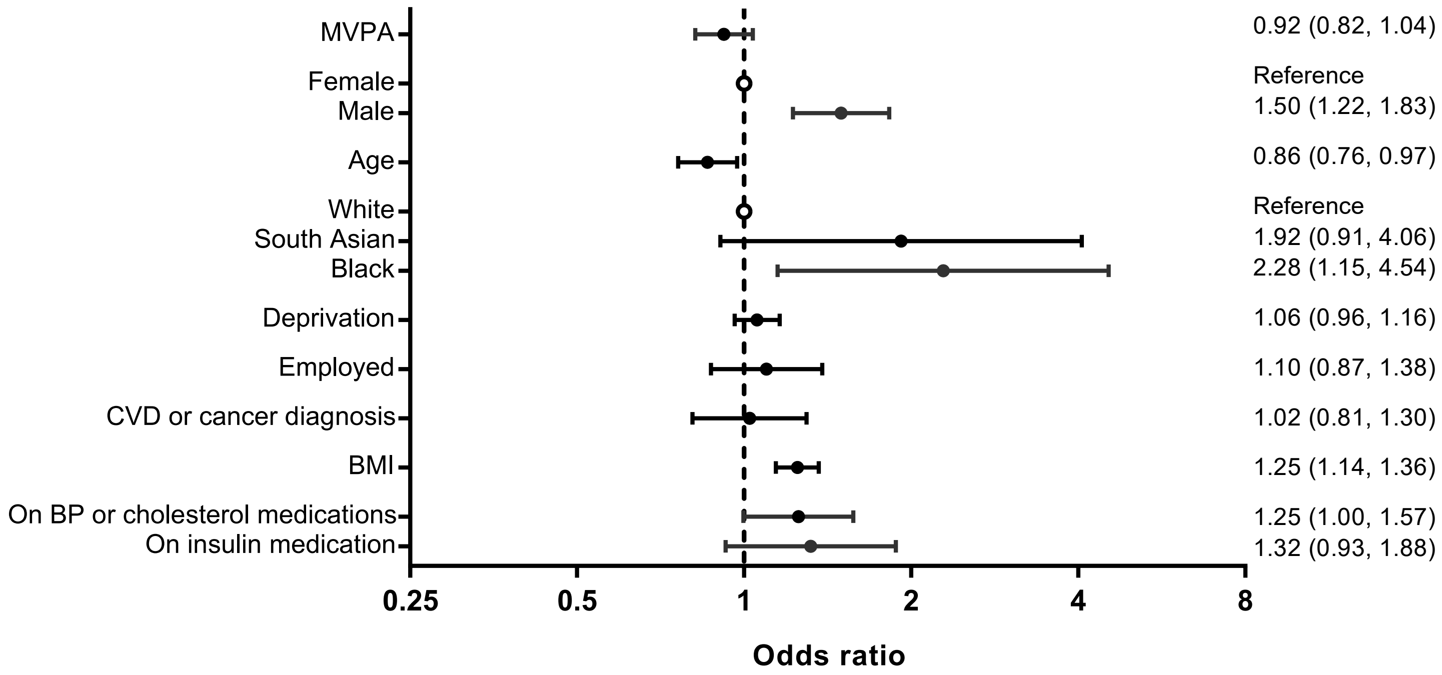

Model 3: Non-severe COVID-19 (Negative COVID-19 test as comparator)

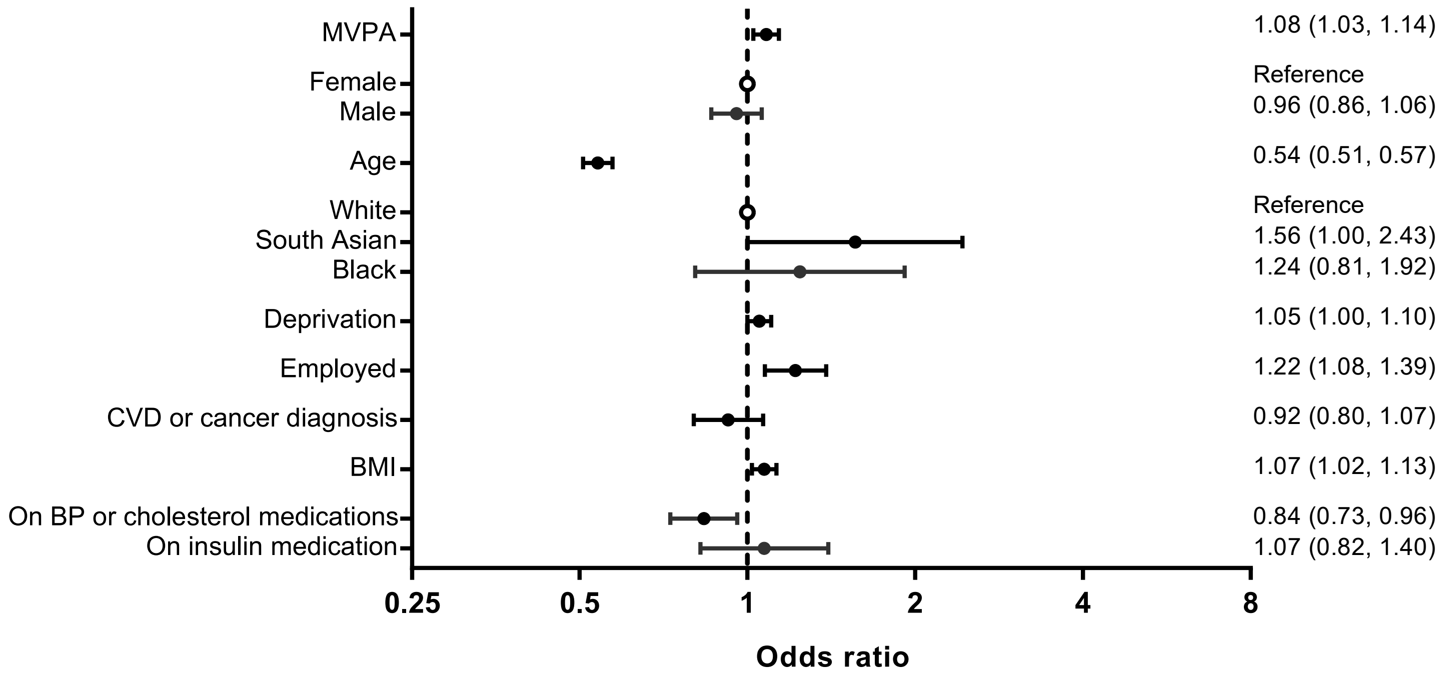

Figure S6. Adjusted odds ratios for risk factors (including health-related co-variates potentially on the causal pathway from physical activity to COVID-19 risk) entered into logistic regression analyses with negative COVID-19 test as comparator and MVPA as the exposure (sensitivity analyses of Models 1 and 3). Continuous variables (MVPA, age, BMI and deprivation) are standardized, thus odds ratios expressed per standard deviation. The adjusted odds ratios for co-variates were similar across all physical activity outcomes. Open circles = reference category.
